# Supplementary material for: COmplexome Profiling ALignment (COPAL) reveals remodeling of mitochondrial protein complexes in Barth syndrome
Source: Bioinformatics. 2019 Jan 14;35(17):3083–91. doi: 10.1093/bioinformatics/btz025 (PMC6735710; doi:10.1093/bioinformatics/btz025)
Supplement: btz025_Supplementary_Materials [file btz025_supplementary_materials.zip › btz025-suppl_data/btz025_Supplementary table 1,4, Figures, Methods_revised.docx]

| **BTHS patient** | **Mutation** |
| --- | --- |
| TAZ001 | c.153C>G |
| TAZ002 | c.239-1G>A |
| TAZ006 | c.170G>T |
| TAZ013 | c.110-1G>C |

**Table S1.** *The four TAZ mutations examined in this study.* Patients were diagnosed with Barth Syndrome (BTHS) based on (ML)CL analysis (Houtkooper et al., 2009). Patient 1 has nonsense mutation, patient 6 has a missense mutation, patients 2 and 13 have a mutation in splice acceptor sites of exon 3 and exon 2 respectively.


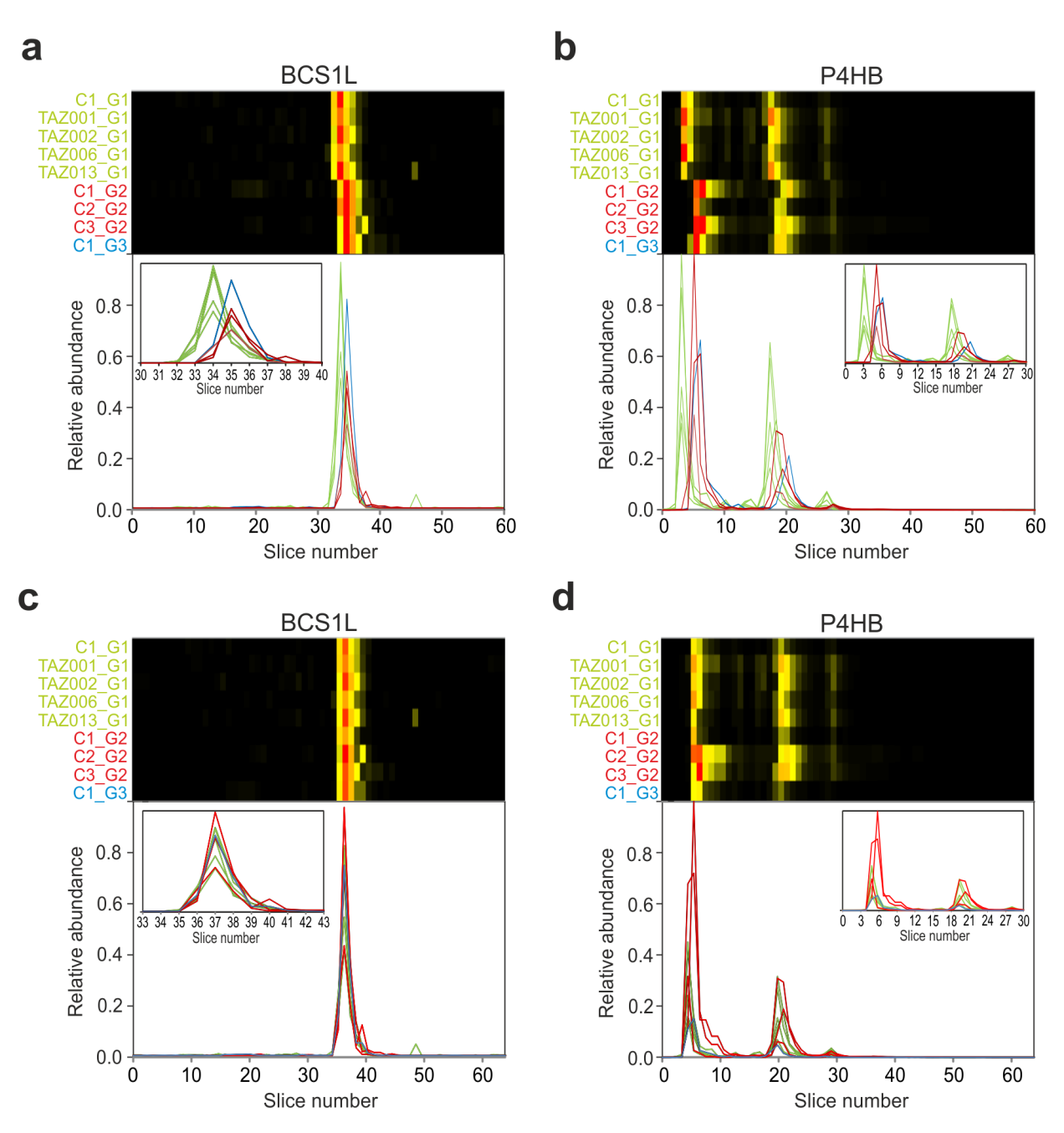


**Figure S1.** *Migration patterns of the P4HB and BCS1L proteins before (a, b) and after (c, d) COPAL alignment.* The color code of the gels is the same as in Figure 2. Slices inserted in the complexomes, based on the migration profiles of all mitochondrial proteins, correctly align the peaks of two individual proteins. The insets zoom into the region of the aligned peaks.


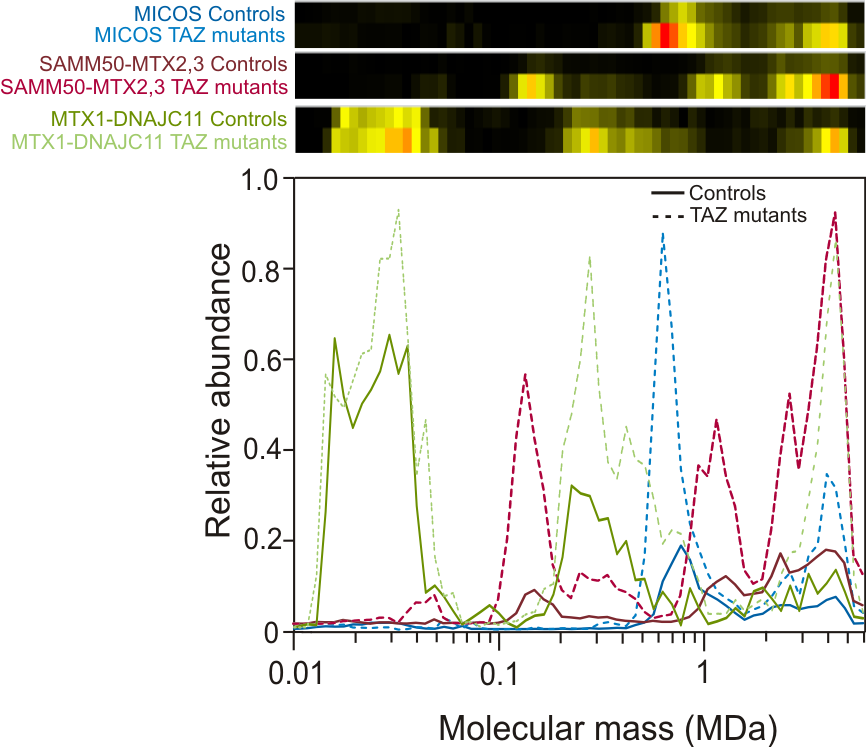


**Figure S2.** *Migration of MIB complex components in control and TAZ mutation fibroblasts.*

The protein subunits of the mitochondrial intermembrane space bridging complex (MIB), consisting of MICOS, SAM (MTX2,3 and SAMM50), MTX1 and DNAJC11, exhibit an overall increase of expression in BTHS mitochondria relative to control. The peaks at ~2.2 kDa and ~4.5 kDa likely correspond to a monomer and dimer of the MIB complex.


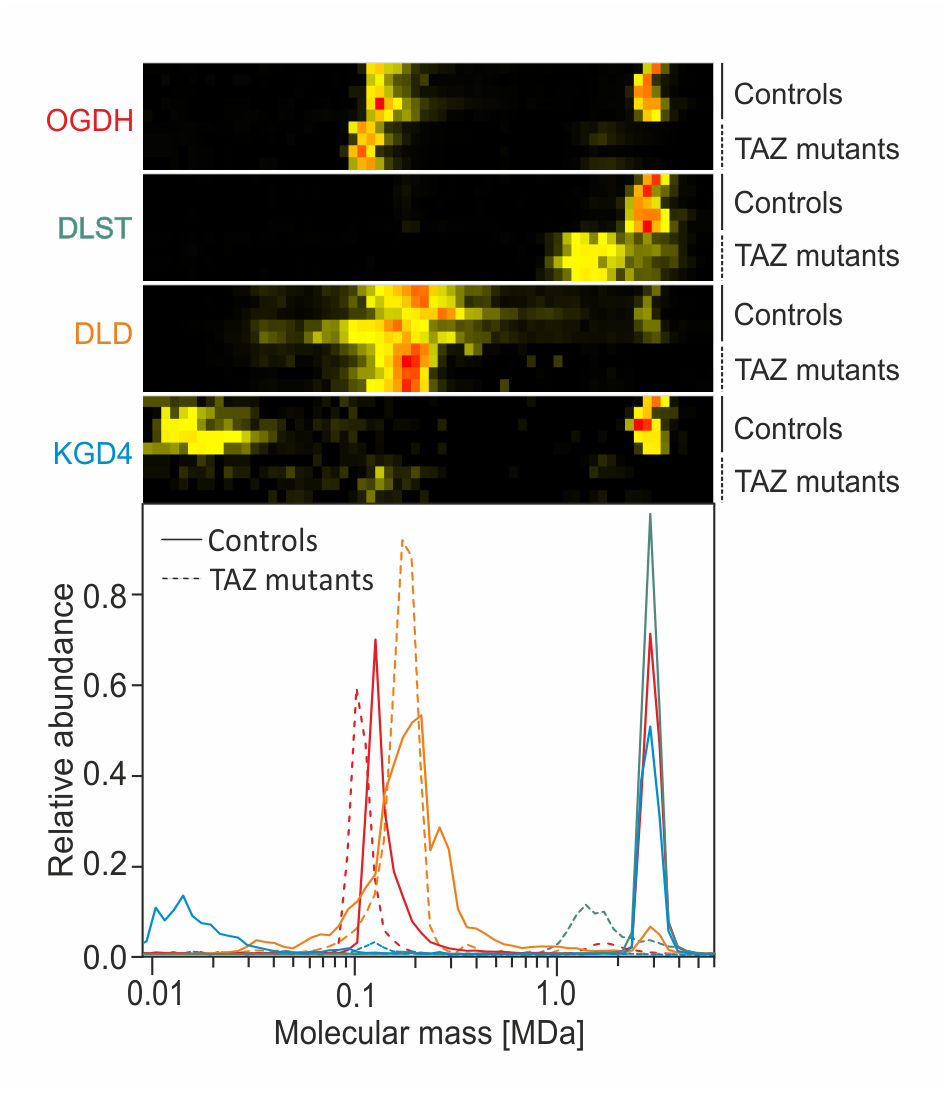


**Figure S3.** *2-oxoglutarate dehydrogenase complex is disassembled in TAZ mutation fibroblasts.*

The 2-oxoglutarate dehydrogenase complex, consisting of dihydrolipoyllysine-succinyltransferase (DLST), dihydrolipoyl-dehydrogenase (DLD), oxoglutarate dehydrogenase (OGDH) and KGD4 (MRPS36) (Heublein et al., 2014), is markedly affected by the mutations. The 3 MDa complex was essentially absent in mitochondria from the BTHS fibroblasts. Instead small amounts of two complexes at ~2.0 MDa containing DLST and OGDH and at ~1.5 MDa containing DLST were observed. While DLST was only detectable in these two large complexes, a similar large reservoir of trimeric DLD (~150 kDa) and monomeric OGDH (~110 kDa) was present in both control and BTHS mitochondria.


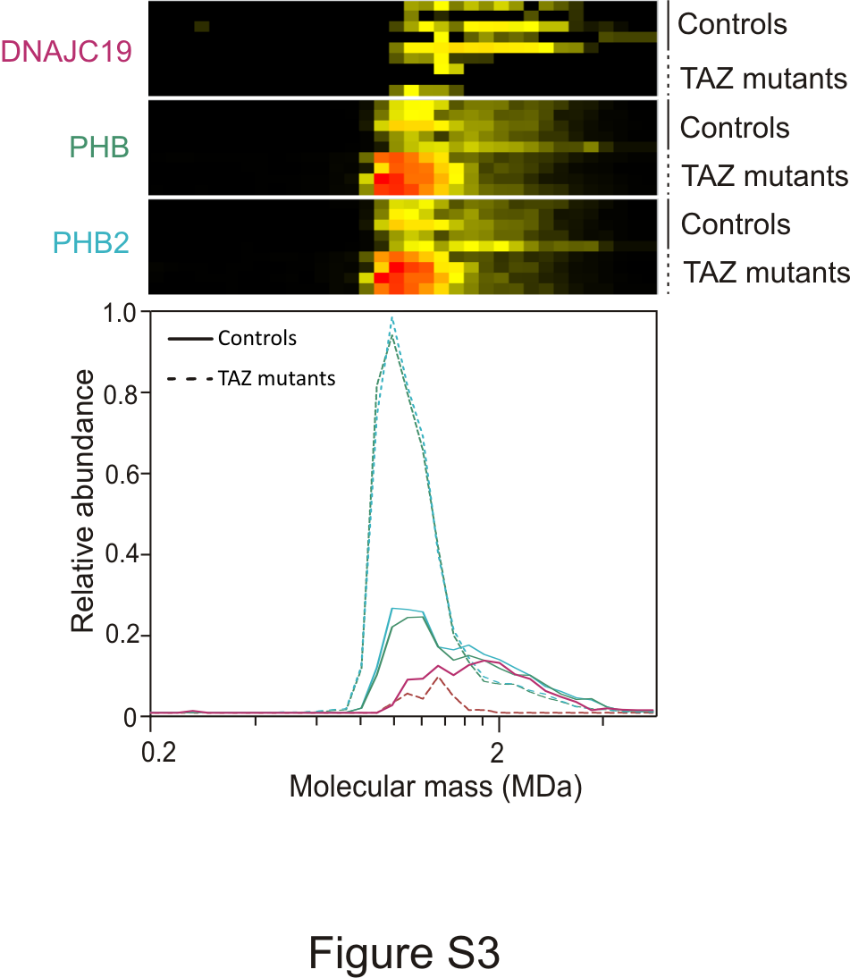


**Figure S4.** *Significant effects of the TAZ mutation on prohibitin.*

The prohibitins (PHB, PHB2) forming a complex of ~1.2 MDa exhibited a ~4-fold increase in abundance while a shoulder in the range from 1.8 - 4.0 MDa was slightly reduced. This corresponded to marked decrease in the abundance of DNAJC19 in the same mass range. Prohibitin is known to take part in CL remodeling in association with DNAJC19 (Richter-Dennerlein et al., 2014).

**
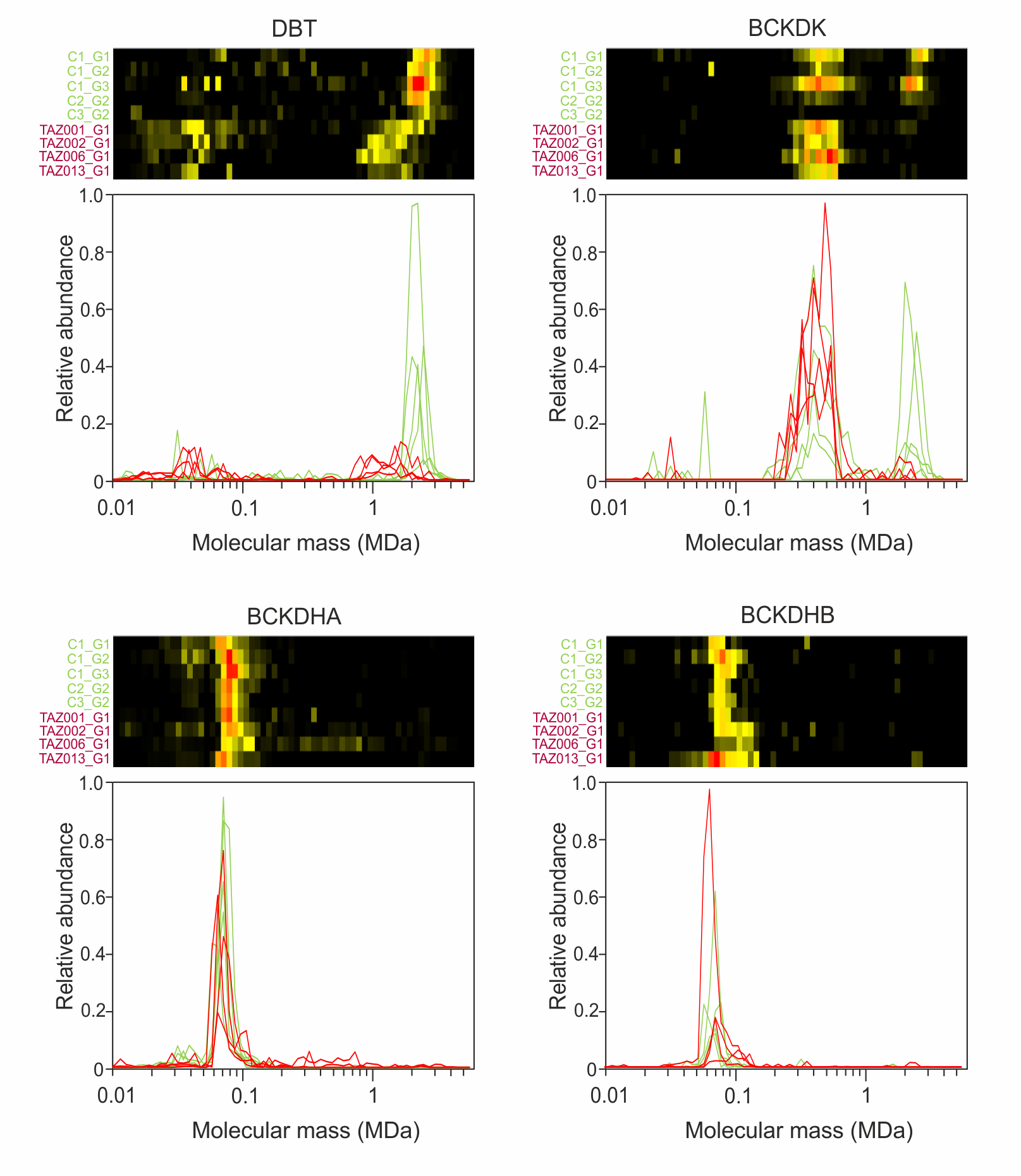
**

**Figure S5.** *Large variation in the presence of the branched-chain amino acid dehydrogenase*

The branched chain dehydrogenase that was found to be disassembled (loss of the peak at ~3 MDa) in BTHS mitochondria compared to one control Chatzispyrou *et al*. (submitted) was not detected as significantly changed in the automated analysis using COPAL and five controls. Closer inspection of the results showed that none of the individual components obtained a high Hausdorff score (Table S3) because there was a large variation in the presence of the ~3 MDa (DBT and BCKDK, upper panels) assembled complex among the controls and because the dominant peak remained at the same mass (BCKDHA, BCKDHB).

**Supplementary methods. The CORUM database**

Manual inspection of the human mitochondrial protein complex list in CORUM revealed a few known complexes that were absent from “human” complexes in the database but that were present in other mammalian species. These were added to the list used: 2-oxoglutarate dehydrogenase complex, cytochrome *bc_1_*-complex, F_1_F_O_-ATP synthase - IF1 (inhibitor protein) complex, respiratory chain supercomplex (complex I, III, IV) and succinate dehydrogenase (complex II). Furthermore, we added MIC12/QIL1 to the MICOS complex, MTX3 to the MIB complex (Huynen et al., 2016) and we adapted the mitochondrial ribosome subunits based on recent literature (Amunts et al., 2015; Greber et al., 2015).

**References supplementary material**

Amunts, A., Brown, A., Toots, J., Scheres, S.H.W., and Ramakrishnan, V. (2015). Ribosome. The structure of the human mitochondrial ribosome. Science *348*, 95-98.

Greber, B.J., Bieri, P., Leibundgut, M., Leitner, A., Aebersold, R., Boehringer, D., and Ban, N. (2015). Ribosome. The complete structure of the 55S mammalian mitochondrial ribosome. Science *348*, 303-308.

Heublein, M., Burguillos, M.A., Vogtle, F.N., Teixeira, P.F., Imhof, A., Meisinger, C., and Ott, M. (2014). The novel component Kgd4 recruits the E3 subunit to the mitochondrial alpha-ketoglutarate dehydrogenase. Mol Biol Cell *25*, 3342-3349.

Huynen, M.A., Muhlmeister, M., Gotthardt, K., Guerrero-Castillo, S., and Brandt, U. (2016). Evolution and structural organization of the mitochondrial contact site (MICOS) complex and the mitochondrial intermembrane space bridging (MIB) complex. Biochim Biophys Acta *1863*, 91-101.

Richter-Dennerlein, R., Korwitz, A., Haag, M., Tatsuta, T., Dargazanli, S., Baker, M., Decker, T., Lamkemeyer, T., Rugarli, E.I., and Langer, T. (2014). DNAJC19, a mitochondrial cochaperone associated with cardiomyopathy, forms a complex with prohibitins to regulate cardiolipin remodeling. Cell Metab *20*, 158-171.

Subramanian, A., Tamayo, P., Mootha, V.K., Mukherjee, S., Ebert, B.L., Gillette, M.A., Paulovich, A., Pomeroy, S.L., Golub, T.R., Lander, E.S., et al. (2005). Gene set enrichment analysis: a knowledge-based approach for interpreting genome-wide expression profiles. Proc Natl Acad Sci U S A *102*, 15545-15550.

Table S4) Mitochondrial proteins sorted by the Hausdorff effect size of a mutation in the taffazin gene on their abundance profile. See main text for details.

geneID Symbol Hausdorff effect size

37693993 NME3 5,698269487

42476281 VDAC2 5,545355702

48255924 NDUFV3 5,04861963

4507879 VDAC1 4,984911691

4505773 PHB 4,92687829

154354966 IMMT 4,59386202

8923390 CHCHD3 4,491541016

22202629 AIFM1 4,444893553

221307584 PHB2 4,441212599

157388993 SYNJ2BP 4,181426523

13129148 APOO 4,079813398

61743952 AGPAT5 4,018977422

40807491 ACSL1 3,898963304

13027606 MRPL34 3,794394658

25188179 VDAC3 3,765905721

19718741 OSBPL1A 3,610092226

222136639 MTHFD1 3,565611237

28872734 MRPL43 3,520692071

224831243 OPA1 3,462036089

62244044 OCIAD2 3,440127521

156105685 ABCB8 3,409320726

19923748 DLST 3,321998369

21361497 ACAD9 3,301940088

7661806 MRPL15 3,295692041

21265070 MRPL2 3,276890602

7657347 MTCH2 3,046349164

154354962 IMMT 2,948902832

188497754 HK1 2,877320611

323276668 MINOS1 2,871036321

48526509 TIMM50 2,866989413

259013537 DTYMK 2,793277466

49169828 TOMM5 2,790566535

156104864 ACOX3 2,65437332

285002233 GPD2 2,626824726

281604151 PGAM5 2,623024178

186928854 MRPS31 2,590513314

31563366 MRPL55 2,589550244

4758714 MGST3 2,568657415

14165270 MRPL13 2,533693851

45387955 C19orf70 2,531946514

5729937 MTX2 2,525987211

31652226 MRPL21 2,511714286

38505218 ACAD11 2,511145377

32698876 CMC1 2,501802923

333470683 MRPL45 2,498901258

225543166 SAMM50 2,463099397

117168279 MGARP 2,425518702

4757732 AIFM1 2,416757658

7705704 GSTK1 2,305584622

149193319 GRSF1 2,301245964

13027604 MRPS34 2,297998013

4506001 PPOX 2,292038833

21265080 MRPL18 2,279140122

27886582 MRPL39 2,229405238

169636418 MRPL38 2,192239238

56699456 ISCU 2,165575258

7524346 AK2 2,158191979

38016911 STOM 2,139745602

268370293 VARS2 2,117446823

4502987 COX7A1 2,087716194

22547134 MRPL37 2,079076282

5174723 TOMM40 2,068577487

194097323 ECHS1 2,0671733

4758504 HSD17B10 2,042770792

223468571 MRPL23 2,029085422

13376717 OPA3 2,024908878

96975135 RAB24 2,021678568

6912482 LETM1 2,020883856

4557303 ALDH3A2 2,011397857

261862352 SHMT2 2,009578668

7661910 EMC2 2,007228771

51243059 MCAT 1,984742024

300192933 AFG3L2 1,9801032

21265096 MRPL50 1,979977409

116008154 LYRM9 1,965087842

160420328 ISCA2 1,962979025

116812610 APOOL 1,947796577

45237193 SLC25A44 1,946440179

4758484 GSTO1 1,945309237

38569423 ACLY 1,943222556

169808399 PDSS2 1,929321163

282396088 COA7 1,927573911

13376617 PTGES2 1,914375448

190194365 MTPAP 1,911079897

4557032 LDHB 1,894732444

4505361 NDUFB3 1,875084369

29789409 NDUFAF2 1,865014914

385648253 SPATA20 1,856613563

260166619 ELAC2 1,844584238

4501853 ACAA1 1,843849565

44680136 BDH1 1,835768389

59710109 TIMMDC1 1,834680831

298231213 DNAJC19 1,832818732

75750476 RHOT1 1,830846001

10092689 LYRM2 1,830356335

149408153 FAM210A 1,825508862

22547138 MRPL4 1,821260386

4502125 NUDT2 1,814696223

41872631 FASN 1,801674894

24308295 GRPEL1 1,798163861

94536771 COA3 1,785988578

171184400 ABCB10 1,785846416

171846268 TSFM 1,782095093

148728172 HTATIP2 1,774001855

26638659 MRPL40 1,763226475

4557403 SLC25A20 1,755184579

4507149 SOD1 1,74975123

21687127 CCDC127 1,748656459

58331150 MRPS30 1,748373694

38569475 MTX1 1,747387294

7706333 MRPL48 1,745984128

9945306 MGST1 1,745584536

11545863 MCCC2 1,733872464

5453722 LYPLA1 1,72723719

40217812 TBRG4 1,725184405

91199540 DLD 1,71350395

344179101 ATPAF1 1,711599137

4502209 ARF5 1,707655225

51558774 ATP5S 1,707309359

7705805 MRPS2 1,706494607

117606353 MSRB2 1,704470627

7705851 CHCHD2 1,697770514

116063536 COQ5 1,696085238

62988355 CCDC58 1,695975727

28178821 IDH3B 1,692486594

223718097 OXA1L 1,690921021

9910244 MRPS22 1,687112204

283436222 ATAD3A 1,686355308

13899231 MRPL9 1,685147326

74099704 SUOX 1,67974016

19923437 AK3 1,665054702

21314739 SLC25A32 1,665050724

4758638 PRDX6 1,661575143

42516576 GLRX5 1,660892759

15029520 HARS2 1,656931095

281604136 PGAM5 1,651882845

4502381 BCL2L1 1,649851254

21362030 TOMM40L 1,646934104

7661872 LARS2 1,642575896

14150017 TMEM126A 1,631893659

7242140 CLPX 1,630250211

4503609 ETFB 1,627554457

42544174 DTYMK 1,621149817

51873036 OGDH 1,619871534

21361378 SARDH 1,616851396

31621305 LRPPRC 1,614124886

134142062 ACACB 1,604797479

337756507 MTHFD1L 1,604635662

5031987 PPIF 1,601788133

86198310 SLC25A29 1,599418725

7705630 MRPS18C 1,599347484

5803135 RAB35 1,594906302

31711992 DLAT 1,594458583

281182727 SCO2 1,591800106

145309326 LAMC1 1,589150877

7661672 POLDIP2 1,582192532

91176333 LYRM7 1,58148409

50978624 GATC 1,580680057

8923427 OCIAD1 1,570108805

4758790 NDUFS5 1,568719153

4757860 BNIP3L 1,562398493

11024700 TIMM13 1,554239804

8922625 TMLHE 1,551908805

94557305 MRPL19 1,55190123

165377209 METTL15 1,546792381

70166634 PUS1 1,544416833

4504007 GK 1,540932645

143770802 MRPL1 1,532517629

7705618 MRPL11 1,527037106

23200008 ABHD11 1,523975839

189095269 PCCA 1,518676006

19923233 SCP2 1,518649039

7657345 MTCH1 1,513910165

18152785 ROMO1 1,512858979

10190746 RDH14 1,503131023

189027129 PDE12 1,501402999

189491675 FAM136A 1,500614448

197100773 RARS2 1,49978594

4502491 C1QBP 1,499144784

5032181 TIMM17B 1,496947263

7656849 ACAD8 1,494201539

4505145 ME2 1,49399058

23200012 ABHD11 1,491627966

267844826 MTX3 1,490783864

231567183 ACOT13 1,487916696

15150811 MRPS36 1,486854015

31083077 MRPL52 1,486823091

258645122 SLC25A26 1,486401215

8923421 SARS2 1,485645052

148728170 HTATIP2 1,484574615

4759048 MRPL33 1,482493366

21265093 MRPL41 1,479951387

300795542 SIRT5 1,478600588

7657585 SLC25A15 1,474586219

32528286 ACOT7 1,473964014

392494079 DBT 1,471947049

116812630 SDR39U1 1,469181304

50345988 ATP5C1 1,467956772

343168767 ACSF3 1,466085613

24371248 FUNDC2 1,465049227

16554616 MRPS6 1,463458658

124249392 DNLZ 1,463126511

16117789 RPL34 1,455781076

28872736 MRPL43 1,455077948

385719194 CLPB 1,454244661

49574510 NDUFAF1 1,447544767

49355721 FAM162A 1,441053728

4502327 AUH 1,440119957

71772415 RPS15A 1,434171427

118200356 GHITM 1,432769361

4758332 ACSL4 1,430946225

4504327 HADHB 1,425233779

4502991 COX7B 1,425206764

9845297 DIABLO 1,423628354

51479132 ATP5J2 1,421272298

83627705 UQCRH 1,421073605

359718987 YME1L1 1,414552126

392583865 SLIRP 1,407192679

406601114 TTC19 1,404622914

34147630 TUFM 1,403302599

11128019 CYCS 1,401848133

157694516 ECHDC1 1,400448339

4502985 COX6B1 1,398896443

75750470 RHOT1 1,392480258

50593025 NFU1 1,389585464

83776598 MAVS 1,388575545

333609242 SMDT1 1,388550574

7710154 WARS2 1,38593061

395394071 TST 1,385093884

42558252 CHCHD1 1,384201308

41462412 SLC25A35 1,37891846

321267511 NIPSNAP1 1,378137812

4506341 ABCD3 1,375341329

7706057 MRPL27 1,369986504

26667177 MRPL46 1,368636655

358248211 ACSS1 1,36709762

11559925 XPNPEP3 1,360463033

4826649 MRPL49 1,360238573

13994261 MRPL32 1,354421565

217035105 DNAJC11 1,352952988

4758352 FDX1 1,347399971

222831590 QRSL1 1,346367228

4759154 SNAP29 1,345564564

156071459 SLC25A5 1,344702501

8923448 MRPL16 1,34425203

4503023 CPT2 1,344186837

8922532 RMDN3 1,342328588

5174419 CLPP 1,341935718

16554609 MRPS11 1,338348838

41327741 ETHE1 1,335530302

22035590 MRPL24 1,334371674

31077081 FXN 1,33392451

221316661 OGDHL 1,333333333

255759952 WDR81 1,333333333

16596694 MRPL53 1,331586914

156151445 ACSF2 1,330934729

14269586 MRPS26 1,330301313

7662004 MFN2 1,327606605

7705927 ATPIF1 1,32658378

55769543 MRPL14 1,325791657

94721354 MTHFD2 1,325671408

62912457 ALDH18A1 1,324380063

111955139 GTPBP10 1,319992899

38202207 MAOB 1,319114183

14028877 MRPS25 1,317449156

28557745 ZADH2 1,315662179

341926262 COQ6 1,313177177

302129698 TMEM126B 1,310851318

385648255 SPATA20 1,305222072

7706349 MRPS33 1,304048829

94538320 HAGH 1,298698574

12597661 MRPL44 1,298624256

103472001 NDUFA7 1,296476278

8923892 PXMP2 1,296436117

356874780 NT5C 1,291505697

5031777 IDH3A 1,290126967

110227603 COASY 1,290095104

6912328 DDAH1 1,285705006

296179429 HADH 1,2828341

7661730 MRPS28 1,282309854

13027640 AASS 1,279717057

26638657 MRPL20 1,278307569

51479156 ATP5L 1,275704903

14150070 CHCHD5 1,269884849

32189392 PRDX2 1,268544884

296841095 PACSIN2 1,268271211

4507319 SURF1 1,268204438

17921987 COX15 1,267706059

21265040 MRPL22 1,267376062

20270311 SLC25A46 1,265569723

383209673 CRAT 1,264731823

116875831 RMDN1 1,263543527

21361565 ATP5F1 1,257609132

76159293 THEM4 1,256630509

145275185 C12orf10 1,255137994

78711824 SDHC 1,254919006

4885079 ATP5C1 1,251874884

155969707 IDE 1,2513501

23110944 PSMA6 1,250642843

32483357 DHRS4 1,250291873

191252797 DCAKD 1,250235506

72534754 FDX1L 1,249256767

5031951 NME6 1,249055881

344179096 ATPAF1 1,247185517

157384956 NUBPL 1,242627403

148727286 ACOT2 1,239839482

5453539 PAICS 1,239111727

145580604 TMEM65 1,237333235

148612885 ARL2 1,234718033

94536784 CCDC51 1,233374239

30089974 ACOX1 1,231622396

47458811 SFXN4 1,229893637

29826287 MRPL47 1,22982727

118600965 COQ3 1,229076454

348041302 PI4KA 1,228944525

4503847 SLC37A4 1,225885954

5803133 RAB32 1,223146301

45643119 ECI2 1,216754653

4507645 TPI1 1,2157399

221316588 BPHL 1,215652407

7669492 GAPDH 1,214266646

258645172 BCKDHA 1,212951749

268370295 VARS2 1,209994385

6912238 PRDX5 1,208795147

61835204 MPST 1,20831013

53729339 MTIF2 1,205743459

38683855 PTCD3 1,204685056

20270367 TAMM41 1,203233144

4826852 NDUFAB1 1,200717278

373251164 GLS 1,198387026

83367083 UQCRQ 1,197661397

13654276 QTRT1 1,19671985

41281885 UQCR10 1,192021819

51317370 NDUFA6 1,191999158

75677353 ATAD3B 1,189750695

4505901 TMEM11 1,188980843

45439339 PDP1 1,186633785

4504523 HSPE1 1,184377138

83921614 CYB5B 1,183070997

94538354 PMPCB 1,18263796

193804850 DHRSX 1,181026733

34147498 TMEM70 1,179873406

7662645 MRPS18B 1,178478754

26051229 MRPL28 1,176547069

52630440 FKBP8 1,175775013

296531406 C21orf33 1,172398183

4505355 NDUFA2 1,171445628

31377644 ATAD1 1,171276465

163644321 UQCRFS1 1,171122614

51479141 ATP5J 1,170619051

146134341 NADK2 1,170201107

261878539 EHHADH 1,169644684

31621303 SFXN3 1,166966667

13128970 MRPL57 1,165396173

4557231 ACADM 1,161219146

238776799 ARMC10 1,159258461

157426893 GUF1 1,158828438

205360838 DNAJA3 1,157631493

77404397 SND1 1,156786369

93277094 FAM210B 1,156149907

153085470 HIGD1A 1,155543543

7662637 MRPL42 1,155093385

7705696 TXNDC12 1,154534348

41327764 AKR7A2 1,152471229

117647226 TRMT10C 1,150755012

171906593 BCKDK 1,150097301

4505143 ME1 1,149908986

226371731 HSDL1 1,147485494

384475521 TKT 1,147318094

31542325 CROT 1,146318523

4505367 NDUFC1 1,144059428

21361403 TXN2 1,143596224

4758788 NDUFS3 1,14128779

50345991 ATP5D 1,141133056

11596859 MRPL17 1,140302222

148491091 SLC25A24 1,135104939

13654274 FAHD1 1,133804328

155030240 TRNT1 1,129672692

18105037 COX7A2L 1,128881964

222831587 TRMT61B 1,126771927

4506863 SDHC 1,121119491

37620210 COX20 1,120159966

167614485 ACAA2 1,120065424

157739940 NUDT19 1,116208236

15721937 MRPS24 1,116002178

7657257 TOMM20 1,114572558

25777736 ALDH4A1 1,113257705

50592988 UQCRC2 1,111077611

4758768 NDUFA10 1,110686914

16950607 MRPL51 1,107784176

106049528 PC 1,104650523

41393599 CPOX 1,103507281

40385867 METAP1D 1,101819704

21735485 ATPAF2 1,101806343

19743875 FH 1,100140587

55770836 SCCPDH 1,099898107

4502601 CBR3 1,099865903

48476342 SLC25A23 1,099776989

11968182 RPS18 1,097326557

20270341 LYPLAL1 1,095877668

209862901 DHRS1 1,095135883

16554604 MRPS23 1,095119586

156416003 SDHA 1,094173289

9910184 CMC2 1,093926167

46409324 SPRYD4 1,093369721

20127408 HADHA 1,0932541

5453559 ATP5H 1,092614585

122939155 NNT 1,091497188

119943100 PCCB 1,089657899

260656005 NDUFV1 1,089463493

19923977 MALSU1 1,089444795

4505719 PEX11B 1,089154666

13375695 CARS2 1,088917981

31543415 GADD45GIP1 1,086326976

14150134 CHCHD6 1,085393805

25777608 NLRX1 1,084197478

156071462 SLC25A6 1,084110546

209862760 NDUFB11 1,083877481

334085252 PITRM1 1,081117511

33636719 TIMM44 1,081105056

242117893 BAX 1,080690706

56682959 FTH1 1,077186452

16950593 MRPS21 1,077071963

32307132 NFS1 1,076905596

46094065 SLC25A40 1,075886669

40254986 HSDL2 1,075425433

45267832 FASTKD2 1,070708866

21735621 MDH2 1,070159303

336455101 AHCYL1 1,065879582

298358756 ATP5G3 1,06461311

41327781 NDUFAF3 1,064319613

38679950 ABAT 1,064114384

398303805 MCU 1,063292446

186928850 MRPS27 1,062469362

28178825 IDH1 1,061684202

41406084 GPX1 1,060789815

7705626 MRPS16 1,060253229

11321583 SUCLA2 1,060116713

116805340 GARS 1,059069099

21361368 ALDH18A1 1,057781882

27436901 MRPL12 1,055967574

331284144 COA6 1,055343576

24307899 ERAL1 1,054831691

145701028 NDUFAF7 1,054798079

20336290 DHX30 1,05473066

209969695 ISOC2 1,05334325

10092657 NDUFA12 1,053113081

50345984 ATP5A1 1,05111724

4507401 TFAM 1,050783575

285002259 HCCS 1,050586587

4758034 COX11 1,049773268

73089054 MSRB3 1,049745624

4759068 SCO1 1,047439821

24308013 PMPCA 1,047334617

4557235 ACADVL 1,04719121

38788380 DHTKD1 1,046717971

32455262 PRDX5 1,046506598

4505369 NDUFS4 1,046409121

7705793 LACTB2 1,045516298

7657369 NDUFA8 1,044216995

9910460 NIT2 1,044025617

16554611 MRPS15 1,043898248

9910382 TOMM22 1,042623677

134288884 EARS2 1,042332326

21396489 LONP1 1,042297302

306922382 MICU1 1,041931439

20149619 DHRS7B 1,041179895

17017988 COX5B 1,038431087

330340389 USMG5 1,038154336

38569421 ACLY 1,036855706

297632350 NIT1 1,036489859

203098816 PDHX 1,035408903

62198232 HMGCL 1,03474006

6005723 CA5B 1,034003866

171460918 DNM1L 1,033571493

7705425 MRPS17 1,03231632

67078404 MECR 1,032052662

4504221 GUK1 1,031750761

5453549 PRDX4 1,030640245

217272839 CHDH 1,030614489

15149476 RARS 1,030489547

304571975 DCXR 1,030310757

21687102 DNAJC19 1,029711558

7262393 ABCD1 1,029105012

4506457 RCN2 1,028660317

6041669 NDUFB4 1,028491715

4758770 NDUFA1 1,028306508

21389351 SFXN5 1,027813573

397739033 SLC16A7 1,026935108

115387094 SDHB 1,025582173

5454152 UQCRB 1,024874415

31377607 MFF 1,024513401

156231349 FAHD2A 1,023390028

32189394 ATP5B 1,022735116

8923708 FOXRED1 1,022054437

4885287 GNG5 1,020518322

283837866 PET100 1,020143973

6912396 GRHPR 1,019662409

27436908 MRPL54 1,019605301

156105689 MUT 1,019260579

47132595 SLC25A3 1,018793164

188528628 PNPT1 1,017901521

57164948 SLC30A9 1,017383892

157779135 SUCLG2 1,016711713

28178832 IDH2 1,015008837

40018633 IDI1 1,014790242

4502303 ATP5O 1,014357956

56605994 CISD2 1,01428737

153792148 THNSL1 1,014219931

226491502 NARS2 1,014140835

62530384 ECI1 1,011924159

315434216 DAP3 1,011189281

11559927 MRPS14 1,011143429

30089972 ACOX1 1,008787365

22749443 MICU2 1,008402852

148224884 PTPMT1 1,008275684

238814322 ALDH1L2 1,007287432

115387104 ALDH9A1 1,007231073

6005717 ATP5I 1,005967007

27545315 TACO1 1,005616888

37594457 NUDT9 1,005237048

155722983 TRAP1 1,005094544

49169841 C8orf82 1,004238789

7657202 HSPB7 1,003461633

7305503 STOML2 1,002483119

70906441 DUT 1,001587233

116235468 MPV17L2 1,000843741

63055043 TMEM205 1,000071958

70995211 ECH1 0,998427604

296923776 UQCC1 0,997948183

7706351 PTRH2 0,99787471

16950591 MRPS12 0,99776844

21361103 SLC25A12 0,997528793

41349497 PDP2 0,996534649

30578418 SFXN2 0,995909196

7657581 SLC25A13 0,995705674

194306651 PDPR 0,994323738

33519430 TXNRD1 0,993950198

37694067 C15orf48 0,993457891

7330335 CLIC4 0,992091657

56605983 NIF3L1 0,99077362

31543667 SUPV3L1 0,989819237

226874831 GUK1 0,989236814

57863271 VWA8 0,989027805

5803217 UQCR11 0,986631441

305410789 GSR 0,986181808

46593007 UQCRC1 0,985551671

332635095 PLGRKT 0,984954181

42490749 ABCB7 0,984897265

33519475 NDUFS1 0,983506112

68051721 NCEH1 0,98342007

156142176 TIMM21 0,982866429

8923001 ABHD10 0,982125921

186928852 MRPS7 0,978554821

251831110 COX2 0,978541208

20336761 HEBP1 0,978345534

148612859 CHCHD4 0,978136765

22538497 DNAJC30 0,977574685

5454120 TIMM17A 0,975608096

13994259 MRPS5 0,975390443

73623030 CPT1A 0,975196648

5454122 TIMM23 0,974534445

251831111 ATP8 0,973913461

365192581 PNPLA8 0,972527266

5031815 KARS 0,972057955

4826854 NDUFB8 0,97186692

31543380 PARK7 0,97132971

108773799 GDAP1 0,970160274

5901896 ATP5E 0,968812398

47519420 RTN4IP1 0,968718859

4557735 MAOA 0,966451869

4501859 ACADSB 0,966436246

4758772 NDUFA3 0,966169496

4505685 PDHA1 0,966088997

21361356 TOMM34 0,965963181

261399877 CCT7 0,965784452

4504505 HSD17B4 0,96565534

6912660 SIRT3 0,965557107

13376331 L2HGDH 0,965397915

7661786 NDUFAF4 0,964983313

20070344 TARS2 0,964130903

4758774 NDUFB10 0,964059998

197116386 NT5DC2 0,963897328

111118981 FDXR 0,962594782

14249680 PYURF 0,96212795

51243057 MTFP1 0,961661719

386642860 TARS 0,961439389

11095441 ALDH6A1 0,961082474

6005862 MRPL3 0,96087011

7661602 MPC2 0,960714399

8051579 AK4 0,960668538

11545859 GOLPH3 0,959409169

251831116 ND4 0,958835657

4557237 ACAT1 0,958784365

94420683 TRMT11 0,958521252

217330598 GLOD4 0,956084682

20149598 SLC25A10 0,955394282

4758582 IDH3G 0,955182094

21361114 SLC25A11 0,955013421

300796991 SLC25A22 0,954757847

18201905 GPI 0,954488536

8923930 CISD1 0,95441671

6552328 CYB5R3 0,953937391

6681764 NDUFA9 0,95305618

4758786 NDUFS2 0,949521337

24797097 PYCR1 0,948936453

319655561 ALDH7A1 0,948825856

194239674 CRYZ 0,948218999

38570054 LYRM4 0,948194117

7669481 BNIP3 0,948010912

4502363 BAK1 0,94656411

50592996 TUBB3 0,946502639

4503607 ETFA 0,946391331

13654294 HDHD3 0,945485676

72534716 MCUR1 0,944840903

27363461 PAM16 0,944724451

56606061 TIMM22 0,943704317

14150066 ALKBH7 0,943360397

284172438 PREPL 0,943184601

45269137 MFN1 0,942581408

13375727 ACSS3 0,941673136

94681057 YARS2 0,940504219

41399285 HSPD1 0,939079086

290656975 TK2 0,938916059

37594471 HIBCH 0,937972472

17999528 COX6A1 0,9377405

51243055 MTFP1 0,936234067

4758668 SPTLC2 0,936056901

19923959 SDSL 0,935103832

109452591 SUCLG1 0,934547415

7705501 TMEM14C 0,932256603

224451073 EXOG 0,932198273

50658084 BCAT2 0,932093999

5174539 MDH1 0,931035865

16418349 MMAB 0,929827065

14149738 NLN 0,929366506

8922498 PNPO 0,9279819

4557014 CAT 0,92607452

166795268 RDH11 0,924937872

20070125 P4HB 0,924255604

4505357 NDUFA4 0,924230775

4503301 DECR1 0,923283047

251831112 ATP6 0,921676819

25777732 ALDH2 0,921165819

72534692 NT5DC3 0,921001837

29171702 PPA2 0,920532944

67782307 SOD2 0,919311492

14211923 HINT2 0,918621041

4758784 NDUFC2 0,917656885

226958412 IVD 0,917398075

74275350 TSPO 0,917282904

38327625 CS 0,917140355

33188463 MRPS9 0,91685265

119964728 D2HGDH 0,916649619

4557817 OXCT1 0,916286319

21361331 CPS1 0,915948305

21359867 CYC1 0,915604618

4503211 CYP27A1 0,915396499

7706029 C6orf203 0,91364083

19924145 TCIRG1 0,913438813

73486658 GOT2 0,912415388

21735604 MRPL30 0,912043673

251831108 ND2 0,911832737

4502993 COX7C 0,911051337

8922511 MRPS18A 0,910101778

156139135 C14orf159 0,909062766

20270317 MRRF 0,908540129

4826862 NME4 0,908466124

13375618 DHCR24 0,908339871

20149663 CCDC90B 0,907068606

4507231 SSBP1 0,906203368

38569477 MTX1 0,906032117

21396487 NDUFAF7 0,905313082

151108473 FIS1 0,90442748

13569840 MACROD1 0,904389655

256773275 DDX28 0,904336085

48976061 ACAD10 0,904134541

116805327 MCCC1 0,903780349

37594464 NUDT5 0,90376367

20127454 ATIC 0,903077787

8922701 AGK 0,902460714

4885413 HINT1 0,902070741

9506859 TOMM7 0,902036832

38569473 NDUFB1 0,901255899

251831118 ND6 0,901153016

338797808 FDPS 0,90114173

21361454 PYCR2 0,900810588

251831119 CYTB 0,900345066

311893396 ATP5J2-PTCD1 0,899311116

188035928 MCEE 0,899009046

25306287 GFM2 0,898665752

40018642 NDUFAF5 0,898115812

56699401 SLC25A25 0,897073948

38569417 AARS2 0,896074426

4503943 GCDH 0,895115015

24308075 RAB11FIP5 0,893367705

4507185 SPR 0,893217529

22035592 MRPL35 0,89252703

20149633 ECSIT 0,89220435

4502981 COX4I1 0,891166375

22267436 NIPSNAP3A 0,890677193

81295404 ACOT9 0,890414494

6466450 COMT 0,8896994

20270303 RHOT2 0,889661666

40789249 DARS2 0,888956629

4758044 COX8A 0,888749784

156105687 MIPEP 0,887425255

28269681 NDUFA11 0,886419938

4758792 NDUFS6 0,886075959

46852147 IARS2 0,885430013

21686999 OMA1 0,883648583

6912714 TIMM9 0,883203773

4505371 NDUFS8 0,883188234

156104878 GLS 0,882452012

4557361 BID 0,881555403

4507173 SPG7 0,881437996

311082415 AIFM2 0,880108939

84508631 PGS1 0,879384079

83641878 PARL 0,877967756

296434309 FLAD1 0,876484576

4557233 ACADS 0,875549974

23308751 HIBADH 0,875327088

26051231 LACTB 0,874466005

156564403 PDHB 0,873997055

18426967 DGUOK 0,873977538

316660086 NDUFB5 0,873823397

151301035 STARD7 0,872966603

40789233 COQ9 0,872401042

7019477 HTRA2 0,870605814

4501867 ACO2 0,870366443

4758040 COX6C 0,869567817

4505689 PDK1 0,86900548

68160922 RPS14 0,868833661

41352061 PITRM1 0,868477916

192448443 FKBP10 0,868202968

260763955 NDUFA13 0,86778679

55749577 SLC25A4 0,866975776

15431288 RPL10A 0,866777067

41393561 LAP3 0,866617413

4506865 SDHD 0,865966112

34147522 ADCK3 0,865852831

119703746 ETFDH 0,865145323

4505365 NDUFB6 0,863819816

222080062 NDUFV2 0,863777931

330688443 MAVS 0,862837496

9955963 ABCB6 0,862432665

62420882 ME3 0,86176565

208973246 QDPR 0,858976764

225579078 RDH13 0,857892643

4557809 OAT 0,857629989

53759134 ENDOG 0,85753398

8923559 OXSM 0,856457263

224496106 REXO2 0,856363453

20270389 HIGD2A 0,855896187

4503183 CYB5A 0,855810041

205360989 C2orf69 0,855452797

14150128 UQCC2 0,855166741

22547114 MRPL10 0,855151154

18390331 GFM1 0,854878985

60499021 FECH 0,854505184

24234688 HSPA9 0,853961332

45243501 BCL2L13 0,852326389

68989263 EHHADH 0,852216285

21389315 SLC25A1 0,851531691

190885499 COX5A 0,85141457

7706369 MPC1 0,850205425

4826848 NDUFA5 0,848689012

6274550 NDUFB9 0,848187736

251831109 COX1 0,847791159

4757852 BCS1L 0,847392505

5453918 SLC25A17 0,846922758

258679435 PET117 0,844714576

4505241 MPV17 0,843068774

66346721 PCK2 0,842355958

8659555 ACO1 0,840491814

16950603 MRPS35 0,840290108

284507298 OAT 0,839539158

23618867 SFXN1 0,839482258

4826870 NUCB2 0,839476566

251831114 ND3 0,839436705

251831117 ND5 0,837286531

22035672 TXNRD2 0,836797068

10764847 NDUFB7 0,835805718

157388927 RMND1 0,834793589

262118227 COX7A2 0,834789406

45006951 DHODH 0,834066921

42794625 AMACR 0,833396712

4503719 FHIT 0,832315199

262073007 SLC16A1 0,832188095

5729820 FARS2 0,831275548

25777730 ALDH1B1 0,830855502

187281616 NDUFS7 0,828323674

251831113 COX3 0,825166325

4885281 GLUD1 0,822467833

16554607 MRPS10 0,822352347

21918872 LACE1 0,821259589

4758940 C14orf2 0,814708336

58197556 IBA57 0,81443872

4557353 BCKDHB 0,813573828

110349750 MLYCD 0,812955987

251831107 ND1 0,812196225

13489112 PISD 0,809096512

205360840 DNAJA3 0,804051273

154354964 IMMT 0,800326563

165377202 METTL15 0,800106264

197927201 TOMM6 0,800025615

4502015 AKAP1 0,8

4503685 FDPS 0,8

4505363 NDUFB5 0,8

4505937 POLG 0,8

4507229 ALDH5A1 0,8

4507521 TKT 0,8

4758778 NDUFB2 0,8

5453607 CCT7 0,8

5453884 PHYH 0,8

6912382 TIMM10B 0,8

6912664 SIRT5 0,8

7657033 NT5C 0,8

7657118 GCAT 0,8

7661556 TRUB2 0,8

7705897 AADAT 0,8

8923450 SDHAF2 0,8

8923565 AURKAIP1 0,8

8923812 ACOT13 0,8

10190702 MRS2 0,8

11141903 FASTKD5 0,8

11641243 PDF 0,8

14150082 SLC25A33 0,8

15277342 HSD17B8 0,8

15529972 SLC25A51 0,8

16117791 RPL35A 0,8

16506297 MGME1 0,8

19923640 GFM2 0,8

22202624 GSTZ1 0,8

22547189 SHMT1 0,8

24308436 MARS2 0,8

27363457 ADCK4 0,8

27477115 ABCA9 0,8

27544933 SLC25A16 0,8

31083060 MRPL52 0,8

31542641 TRMU 0,8

31542713 MARC2 0,8

32171254 TMEM143 0,8

34303926 PARS2 0,8

40068479 MRM1 0,8

41327685 UQCC1 0,8

45545437 CLYBL 0,8

47778923 CYB5R2 0,8

48255926 NDUFV3 0,8

50301234 HSCB 0,8

50301238 GSR 0,8

50345982 ATP5A1 0,8

51479143 ATP5J 0,8

52426768 ISCA1 0,8

54607072 YBEY 0,8

58197562 SLC25A30 0,8

65506442 PCCA 0,8

75709200 GPX4 0,8

89886352 OXLD1 0,8

110618253 POLRMT 0,8

115430227 LYRM5 0,8

116812636 OSGEPL1 0,8

124517691 NDUFAF6 0,8

133922590 ADHFE1 0,8

149193321 GRSF1 0,8

153251771 MTFR1L 0,8

156415992 TFB1M 0,8

157388991 SLC25A36 0,8

166795303 MTRF1L 0,8

171542821 MUL1 0,8

186928860 SLC25A19 0,8

189217863 HINT3 0,8

190194370 LYRM1 0,8

193082993 GTPBP3 0,8

208609993 MSRA 0,8

211057411 CISD3 0,8

215272362 NIF3L1 0,8

221219008 C15orf61 0,8

223468663 AKR1B10 0,8

228008407 SERAC1 0,8

261878564 MTIF3 0,8

268832172 ATP5SL 0,8

285397577 SETD9 0,8

300863126 SUGCT 0,8

304435905 GTPBP6 0,8

309384271 OXR1 0,8

315113868 MTHFS 0,8

334358861 NRD1 0,8

344925834 FAM213A 0,8

358248205 ACSS1 0,8

371123235 NSUN4 0,8

375493516 KIAA0391 0,8

380254499 COX14 0,8

384381468 FDXR 0,8
